# Supplementary material for: Unveiling unconventional magnetism at the surface of Sr2RuO4
Source: Nat Commun. 2021 Oct 4;12:5792. doi: 10.1038/s41467-021-26020-5 (PMC8490454; doi:10.1038/s41467-021-26020-5)
Supplement: Supplementary file 1 — Supplementary Information [file 41467_2021_26020_MOESM1_ESM.pdf]

Supplementary Information for

Unveiling unconventional magnetism at the surface of Sr_2RuO_4

R. Fittipaldi[1,2,†], R. Hartmann[3,†], M. T. Mercaldo[2], S. Komori[4,c], A. Bjørlig[5], W. Kyung[6], Y. Yasui[7,b], T. Miyoshi[7], L. A. B. Olde Olthof[4], C. M. Palomares Garcia[4], V. Granata[2], I. Keren[8,a], W. Higemoto[9], A. Suter[8], T. Prokscha[8], A. Romano[1,2], C. Noce[2], C. Kim[6], Y. Maeno[7], E. Scheer[3], B. Kalisky[5], J. W. A. Robinson[4], M. Cuoco[1,2,*], Z. Salman[8,*], A. Vecchione[1,2], A. Di Bernardo[3,*]

[1] CNR-SPIN, c/o University of Salerno, I-84084 Fisciano, Salerno, Italy

[2]Dipartimento di Fisica "E.R. Caianiello", University of Salerno, I-84084 Fisciano, Salerno, Italy

[3] Department of Physics, University of Konstanz, 78457 Konstanz, Germany

[4] Department of Materials Science and Metallurgy, University of Cambridge, Cambridge, CB3 0FS, UK

[5] Department of Physics, Bar Ilan University, Ramat Gan, 5920002, Israel

[6] Department of Physics and Astronomy, Seoul National University, Seoul, 08826, Korea

[7] Department of Physics, Kyoto University, Kyoto 606-8502, Japan

[8]Laboratory for Muon Spin Spectroscopy, Paul Scherrer Institute, CH-5232 Villigen PSI, Switzerland

[9]Advanced Science Research Center, Japan Atomic Energy Agency, Tokai, Ibaraki 319-1195, Japan

[†] equally contributed to the work

[a] current address: The Racah Institute of Physics, The Hebrew University of Jerusalem, Jerusalem 91904, Israel

[b] current address: RIKEN, Centre for Emergent Matter Science, Saitama 351-0198, Japan

[c] current address: Department of Physics, Nagoya University, Nagoya 464-8602, Japan

*correspondence to: Mario Cuoco (mario.cuoco@spin.cnr.it), Zaher Salman (zaher.salman@psi.ch) and Angelo Di Bernardo (angelo.dibernardo@uni-konstanz.de)

This file includes:

- Supplementary Figures 1-10
- Supplementary Text with further details about muon data analysis and numerical model for orbital loop current phase in Sr_2RuO_4

Supplementary Figures

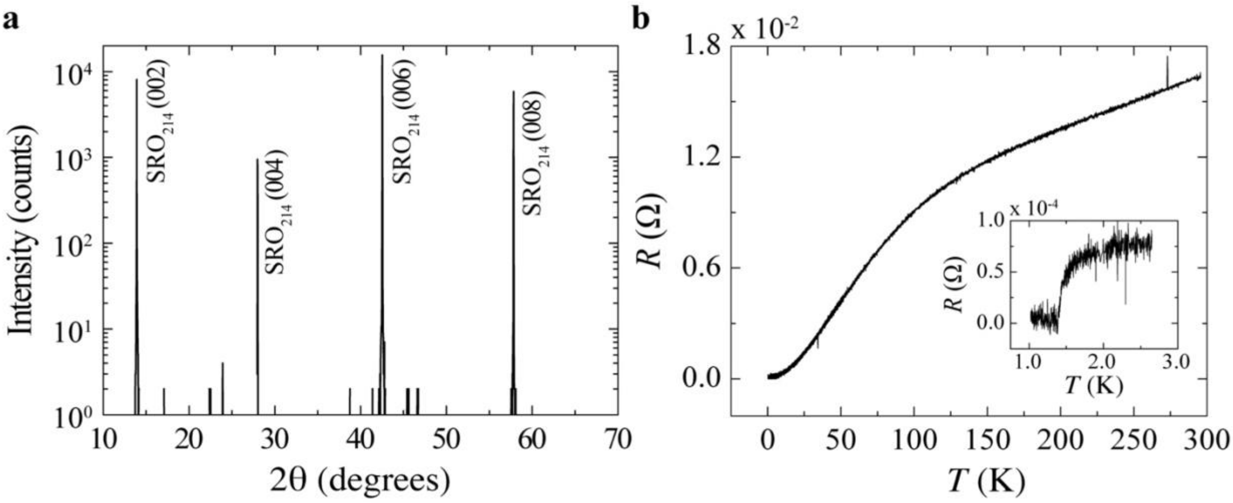

Supplementary Figure 1: Structural and electronic transport properties of SRO_{214}. a, High-angle X-ray diffraction pattern on a SRO_{214} single crystal showing absence of impurity peaks. **b,** Resistance versus temperature curve for a SRO_{214} single crystal measured with current-biased setup in a four-point measurement configuration showing a residual resistance ration larger than 200 and a superconducting critical temperature T_c of ~ 1.45 K (see inset).

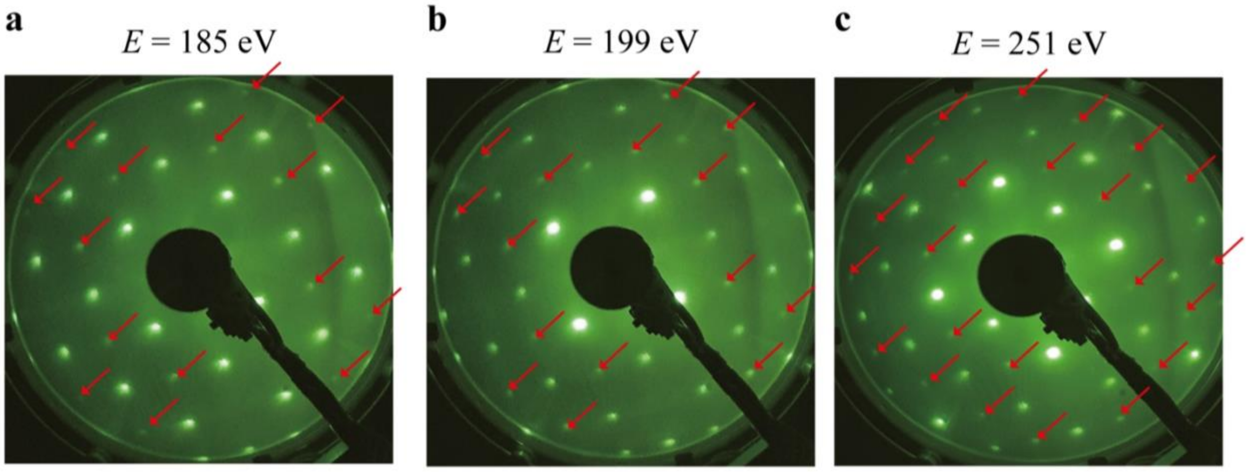

Supplementary Figure 2: Distortion of the RuO_6 octahedra at the SRO_{214} surface. a-c, Low-energy electron diffraction (LEED) pattern acquired on SRO_{214} single crystals at three different energies, E = 185 eV (**a**), E = 199 eV (**b**) and E = 251 eV (**c**) and showing fractional spots (marked by red arrows) which correspond to the distortion of the surface RuO_6 octahedra.

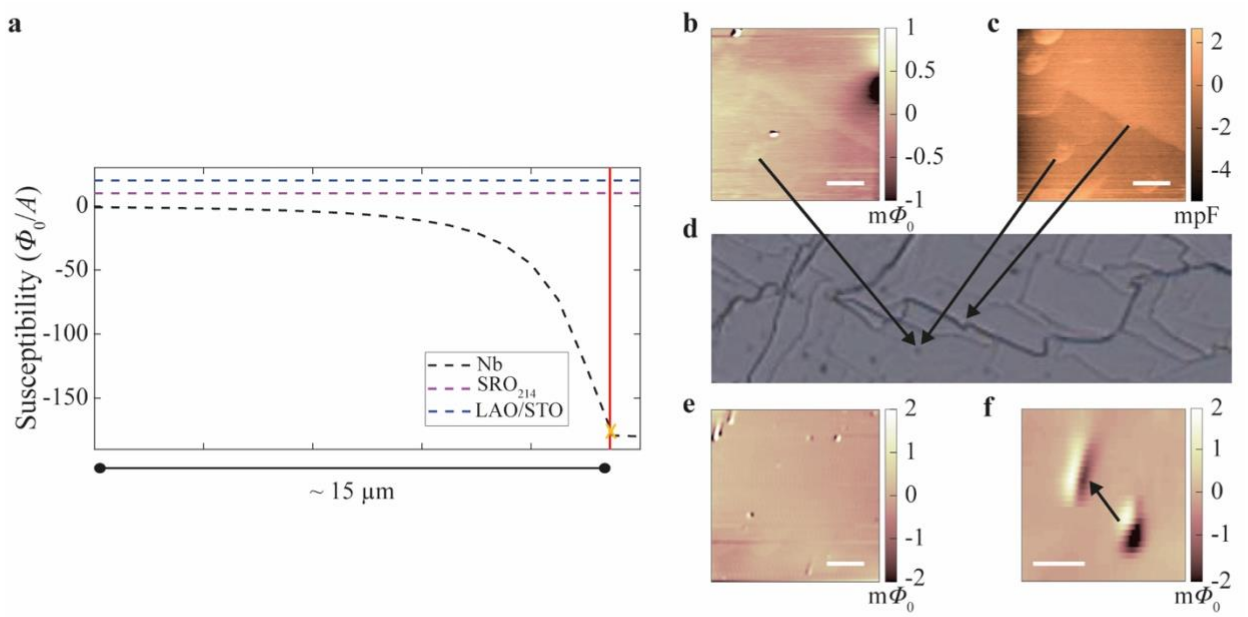

Supplementary Figure 3: Scanning SQUID measurements of SRO_{214}. a, Magnetic susceptibility measured at T = 4.2 K of the SRO_{214} single crystals used for the low energy μSR experiment as a function of SQUID-to-sample distance (bottom axis). The amplitude of the signal measured on SRO_{214} (purple dashed curve) is comparable to that measured on $LaAlO_3/SrTiO_3$ (blue dashed curve) and about 15000 times smaller in modulus than the (diamagnetic) signal measured on a Nb thin film (black dashed curve). We note that all curves begin at 0 magnetic susceptibility but have been offset for clarity. **b**-**d**, Magnetic signal (**b**) and topography (**c**) images recorded on the surface of a SRO_{214} single crystals (**d**) along step edges (in the regions indicated by black arrows in (**d**)). The scale bars in (**b**) and (**c**) correspond to a length of 50 μm. The topography of the SRO_{214} sample combined with the variation in sample-to-SQUID distance can account for the weak signal observed along the sample edges in (**b**). Pillars on the sample – which also induce a variation in the height of the SQUID – give rise to a magnetic signal similar to that generated by edges. **e, f,** Direct current (DC) magnetic map on SRO_{214} showing the absence of any magnetism, except for small spots, most likely extrinsic to the sample and possibly introduced during the cleaving process. These objects can be dragged over the surface (**f**). The scale bars in (**e**) and (**f**) correspond to a length of 50 μm and 10 μm, respectively. The magnetic flux maps in (**b**), (**e**) and (**f**) are shown in units of $m\Phi_0$ (Φ_0 = 2.0678 x 10^{-15} Tesla m^2 being the flux quantum).

Supplementary Text

Further details about the analysis of the low energy μSR data

The experimental setup and corresponding configurations (i.e., transverse, longitudinal and zero field) used to collect the LE-μSR data on SRO_{214} are shown in Fig. 1b, c and explained in the main text of the manuscript.

The starting point for the analysis of the muon data, independently on the configuration used, is the signal called asymmetry $A_s(t)$, which is experimentally determined from Eqs. S1 and S2 below. The number of events N (t, E) is counted at a given time t and energy E by each of the eight positron detectors (i.e., top, bottom, left and right, each consisting of an upstream and a downstream segment) arranged around the SRO_{214} sample, as shown in Fig. 1b of the main manuscript. The number of events recorded by each detector can be written as

$$N^{i}(t) = N_0^{i}\, e^{-t/\tau_{\mu}} \left[1 + A_0 \boldsymbol{P}(t) \cdot \hat{\boldsymbol{n}}_{\mathrm{i}}\right] + N_{\mathrm{Bg}}^{i}, \qquad \text{(Eq. S1)}$$

where the index i refers to a specific detector segment (i = 1, 2, …, 8) and N_0^i and N_{Bg}^i are the number of counts at the initial time $t = 0$ and the number of background counts for the same detector i. In addition, in Eq. S1, A_0 is the asymmetry parameter which depends on the beta-decay symmetry of the muons and on the solid angle formed by the detector segments, **P** (t) is the muons' polarisation, which corresponds to the ensemble average polarization of all muons implanted at an energy E with initial polarization $\mathbf{S}_{\mu+}$ and $\hat{\boldsymbol{n}}_{\mathrm{i}}$ is the unit vector along the direction between the sample and the ith segment of detectors as shown in Supplementary Fig. 4.

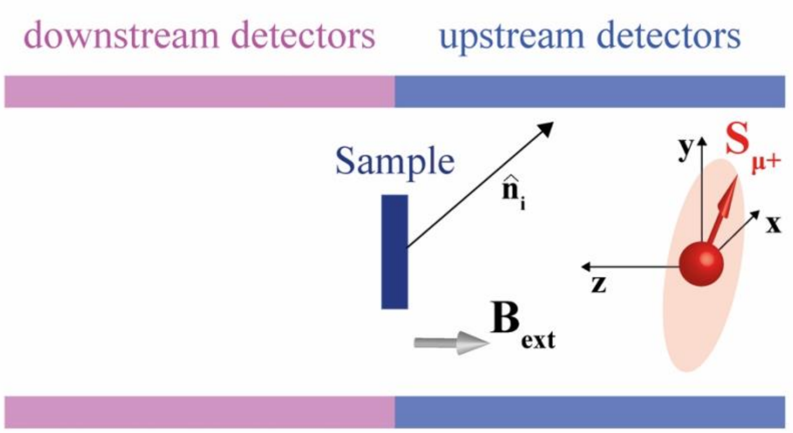

Supplementary Figure 4: Positron detector segment. Schematic showing the arrangement of the investigated sample with respect to one of the detector segments in the low energy μSR measurement apparatus. The number of counts of the detector segment (upstream or downstream) depends on the projection of the muons' spin polarization along the unit vector $\hat{\mathbf{n}}_{\mathrm{i}}$, as described by Eq. S1. The direction of the applied field and the precession plane of the muons refer to the TF configuration used here as an example, but the detector setup is valid independently on the measurement configuration used.

(Eq. S1) also implies that the sum of positron counts of all the upstream or downstream detectors gives information about the projection of **P**(t) along the axis **z**, whilst the sum of the positron counts of the upstream and downstream segments of either the top or the bottom or the left or the right detectors provides information about the projection of **P**(t) in the xy-plane (see Fig. 1b of the main manuscript).

In a transverse field (TF) configuration, we measure the projection **P**(t) in the xy-plane, $P_{xy}(t)$, whereas in a longitudinal field (LF) configuration, we determine the projection of $\mathbf{S}_{\mu+}$ (t) along the direction of the applied external field $\mathbf{B}_{\mathbf{ext}}$ (z-axis in Fig. 1b), i.e., $P_z(t)$. For zero field (ZF) measurements, we measure **P**(t) along the initial muon's spin direction, i.e., left/right in the TF geometry and upwards/downwards in the LF geometry.

In any measurement configuration, the asymmetry $A_s(t, E)$ is weighted average of the muons' spin polarization $P_l(t)$ component, which is determined in that specific configuration, times the muons' stopping depth profile $n_\mu(E, z)$ meaning

$$A_s(t, E) = A_0 \int_0^\infty n_\mu(E, z) P_l(t)\, dz, \qquad \text{(Eq. S2)}$$

where l = z or l = xy depending on the setup used.

Analysis of transverse field (TF) measurements

In TF, the muons are implanted with the initial spin polarization $\mathbf{S}_{\mu+}$ in the xy-plane (nominally along the y-axis) as shown in Fig. 1b of the main paper, and therefore precess in the plane perpendicular to the external applied field $\mathbf{B}_{\mathbf{ext}}$. The time evolution of **P**(t) as the muons are implanted inside the sample provides information about the local field amplitude B_{loc} in the sample, which adds to the external field amplitude B_{ext}, and about the width of the local field distribution. Both B_{loc} and the width of the distribution are averaged over the muons' stopping depth because of the finite width of the muons' implantation profiles n_μ (E, z).

The $A_s(t, E)$ signal determined in TF at a given E best fits to an exponentially damped oscillation according to the relation

$$A_s(t) = A_0\, e^{-\lambda t} \cos\, [\gamma_\mu\, B_{loc}\, t + \varphi_0] \qquad \text{(Eq. S3)}$$

where λ is the muon spin depolarization rate, which is proportional to the width of the local field distribution, and φ_0 is the initial muon phase which depends on the muons' initial spin direction and on the detectors geometry. From the fitting of the experimental $A_s(t, E)$ measured at a given E

to the expression described by (Eq. S3), we determine B_{loc} and λ (averaged over the muon implantation distribution depth), as well as φ_0 and A_0 for each E value.

Examples of $A_s(t, E)$ measured from the positron counts of one detector according to Eq. S1 and corresponding fits to Eq. S3 are shown in Supplementary Fig. 5a. From the fit, we obtain the λ value in Eq. S3, which is related to the time decay of $A_s(t, E)$ as explained above. The Fourier transform of $A_s(t, E)$ is proportional to the local field $p(B_{loc})$ probability distribution, whose mean value corresponds to the local field B_{loc} probed by muons averaged over their implantation depth range at a given E. The raw data for $A_s(t, E)$ and $p(B_{loc})$ in Supplementary Fig. 5a, b and corresponding theoretical fits are used to determine some of the data points shown in Fig. 2 and Fig. 3c of the main text.

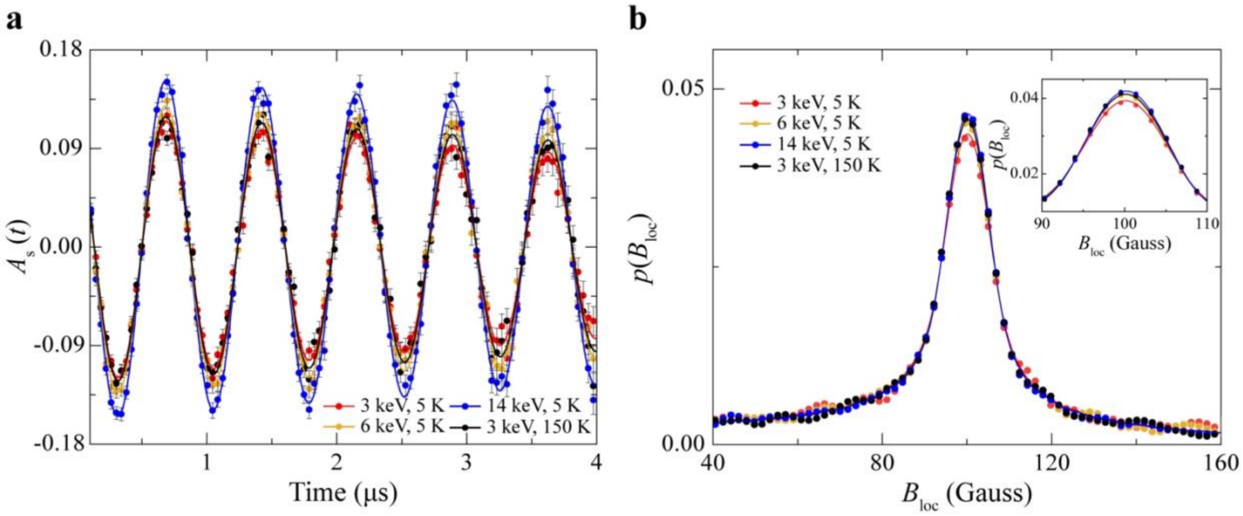

Supplementary Figure 5: Asymmetry signal and corresponding local field distribution. a, b, Examples of raw data (symbols with error bars) and theoretical fits (solid lines) for the asymmetry signal $A_S(t)$ measured in SRO_{214} and corresponding local field probability distribution $p(B_{loc})$ determined from $A_S(t)$ via a Fourier transformation. The representative $A_s(t)$ profiles in (**a**) and relative $p(B_{loc})$ in (**b**) are measured at T = 5 K for E = 3 keV (red), E = 6 keV (light brown), E = 14 keV (blue) and at T = 150 K for E = 3 keV (black). The λ values extracted from the fits of the $A_s(t)$ profiles in (**a**) are used to determine the corresponding data points in Fig. 2 of the main text, and show that λ is higher at low energy (E = 3 keV; red curve) compared to high energy (E = 14 keV; blue curve) due to the surface nature of the magnetism probed in SRO_{214}, and also that λ decreases at higher temperature (i.e., E = 3 keV, T = 150 keV; black curve), where it becomes comparable to λ at E = 6 keV and T = 5 keV (light brown curve), consistently with the data in Fig. 2 of the main text. The data in (**b**) show that the $p(B_{loc})$ distributions are monomodal and that the amplitude values of the average local field B_{loc} extracted from the same distributions do not change significantly as a function of E, as shown by the expanded view on the same data around the distribution peaks reported in the inset in (**b**) and also shown by the data in Fig. 3c of the main text.

We note that, for all measurements done at the same E but at different temperature T (i.e., for a T-scan at fixed E), we can safely assume that A_0 and φ_0 are the same, since these parameters are set by the muon initial spin polarisation which is T-independent. To improve the reliability of the fit and reduce the number of free parameters, we therefore fit all $A_s(t, E)$ spectra which are part of the same T-scan to the function given by (Eq. S3) using common (shared) values of A_0 and φ_0, whilst allowing B_{loc} and λ to vary as a function of T (since they are related to sample properties). As a

result of this fitting procedure, we obtain the λ and B_{loc} values used for the T-scans shown in Fig. 2 and Fig. 3a, b of the main text. In particular, we note that in Fig. 2 of the manuscript we do not report the T-dependence of λ but the T-dependence of the shift in the depolarization rate, $\Delta\lambda(T)$, determined from the λ value measured at T = 270 K. The reason for our choice to show the $\Delta\lambda(T)$ profiles at different Es in Fig. 2 of the manuscript other than the $\lambda(T)$ profiles is because the large differences in the absolute values of λ at low E (e.g., E = 3 keV) compared to the of λ values at higher Es (e.g., E = 6 keV and 14 keV) do not reflect actual changes in the physical properties of the SRO_{214} samples, but they are simply due to a variation in the number of backscattered muons. The $\lambda(T)$ profiles measured at different Es are reported for completeness in Supplementary Fig. 6. It is worth noting that the data in Supplementary Fig. 6 show that λ increases by a factor larger than 3 at E = 3 keV when T is decreased from 270 K down to 5 K.

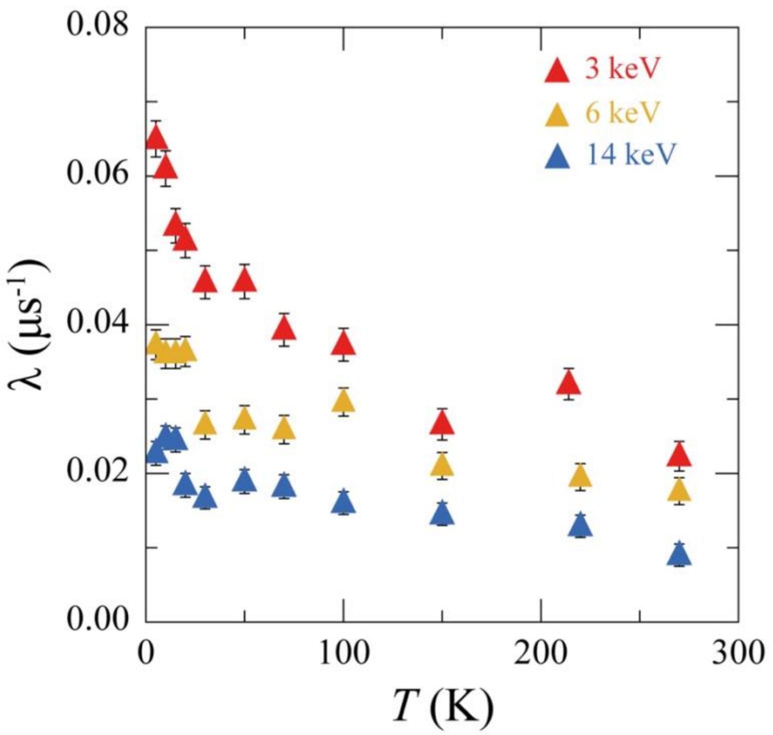

Supplementary Figure 6: Temperature dependence of the depolarization rate in SRO_{214} at different muons' implantation depths.
Depolarization rate λ as a function of temperature T measured in a TF setup with an applied external magnetic field amplitude B_{ext} = 100 Gauss at different implantation energy E values: 3 keV (red symbols with error bars), 6 keV (orange symbols with error bars) and 14 keV (blue symbols with error bars).

For the measurements done at the same T but with varying E (i.e., for an E-scan at a fixed T), we cannot perform a fit using common A_0 and φ_0 to all runs since these parameters are E-dependent. For the E-scans, which are shown in Fig. 3c, d of the main text for two different B_{ext} values, we fit together pairs of runs which are performed at the same E and in the same B_{ext} but at different T (corresponding to T = 5 K < T_{on} and T = 100 K > T_{on} for Fig. 3c, d). The pair of measurements meeting these conditions are fitted together using A_0 and φ_0 as common parameters to both runs, and with λ and B_{loc} as parameters to fit for each individual run.

As explained in the manuscript, from the T-dependence of $\Delta\lambda(T)$ profiles obtained in TF, we also estimate that 50 K < T_{on} < 75 K. This result, which we infer based on the $\Delta\lambda(T)$ profiles shown

in Fig. 2 of the manuscript, is also evidenced by the data sets in Supplementary Fig. 7, where we show the raw asymmetry data and corresponding fits measured at a few representative temperatures (T = 5 K, 50 K and 270 K) at three different energies (E = 3 keV, 6 keV and 14 keV). In particular, Supplementary Fig. 7a shows that the asymmetry signal at E = 3 keV exhibits a significant increase in muons' depolarization from the value measured at 270 K (black curve) already for T = 50 K (light blue curve), as evidenced by the fact that the asymmetry curve at T = 50 K already deviates at a time $t \sim 1.5$ μs from the asymmetry profile at T = 270 K. This is in contrast with the data reported in Supplementary Fig. 7b, c (showing the data for E = 6 keV and 14 keV) where a very small separation between the asymmetry curves at T = 50 K and T = 270 K only becomes visible for a relaxation time larger than 4 μs.

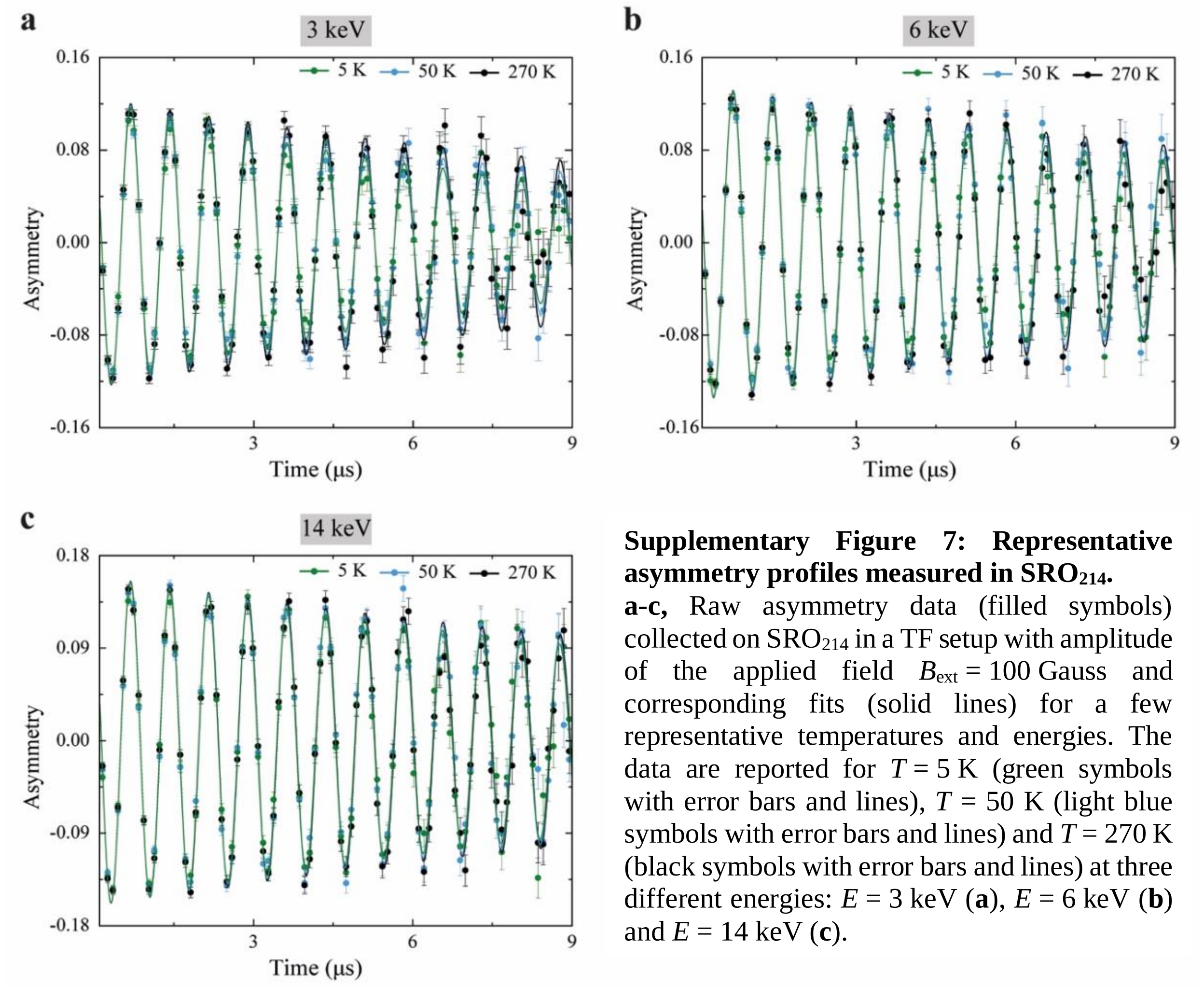

Supplementary Figure 7: Representative asymmetry profiles measured in SRO_{214}.
a-c, Raw asymmetry data (filled symbols) collected on SRO_{214} in a TF setup with amplitude of the applied field B_{ext} = 100 Gauss and corresponding fits (solid lines) for a few representative temperatures and energies. The data are reported for T = 5 K (green symbols with error bars and lines), T = 50 K (light blue symbols with error bars and lines) and T = 270 K (black symbols with error bars and lines) at three different energies: E = 3 keV (**a**), E = 6 keV (**b**) and E = 14 keV (**c**).

The raw asymmetry data therefore suggest, consistently with the $\Delta\lambda$ values extracted from these asymmetry curves and reported for more T values in Fig. 2 of the manuscript, that closer to the SRO_{214} surface at E = 3 keV, the magnetism – which is associated with an increase in the slope of $\Delta\lambda$ – sets in at 50 K < T_{on} < 75 K.

Analysis of longitudinal field (LF) and zero field (ZF) measurements

In the LF and ZF measurements, $\mathbf{B}_{\mathbf{ext}}$ is applied along the same direction (i.e., parallel or antiparallel) as the initial muon spin polarization $\mathbf{S}_{\mu+}(0)$, with the result that the muons do not precess in the plane perpendicular to $\mathbf{B}_{\mathbf{ext}}$ in contrast to the TF configuration. Therefore, the $A_s(t, E)$ signal has no oscillatory component, and it depends on the processes of spin lattice relaxation and depolarization.

At a fixed T and E, we perform two ZF/LF measurements, one with the initial muon spin polarization along the $+z$ and the other along $-z$ (see Fig. 1c of the main text). This approach allows us to increase the signal-to-noise ratio and to avoid systematic errors in the measurements due to changes in the beam optics as result of the muon's spin rotation.

The LF/ZF measurements asymmetry data are then fitted with the theoretical function expected for a Lorentzian static field distribution in ZF and LF (i.e., fit to a static exponential/Lorentzian Kubo-Toyabe function in ZF and LF; ref. [23] of the main text), assuming that the local static fields do not change. In this fit, there is only one free physical parameter which is the Lorentzian field distribution width, meaning that the field dependence (decoupling) is determined by the theoretical function for the corresponding applied field. The fit gives a value of the half width at half maximum (HWHM) of the field distribution of ~ 0.5 Gauss which is consistent with the value that we estimate in the original manuscript for the local static fields probed by muons near the surface of SRO_{214}.

Numerical model for orbital loop current phase in SRO_{214}

To explain the magnetism measured at the surface of SRO_{214} by low-energy muon spin rotation (LE-μSR), we consider an orbital loop current phase emerging at the surface of SRO_{214}. Our theoretical analysis shows that an orbital loop current phase can indeed account for the features of the unconventional magnetism observed (i.e., low magnetic moment of < 0.01 μ_B/Ru atom, high T onset, 50 K < T_{on} < 75 K), whilst being compatible with the translational symmetry of the crystal and with a homogeneous distribution of the magnetism sources over a length scale comparable with the size of a SRO_{214} unit cell.

On the basis of symmetry arguments, we focus on those orbital loop current phases (Supplementary Fig. 8a) that are consistent with the inversion symmetry breaking occurring at the SRO_{214} surface. This consideration leads us to exclude loop current phases of type Θ_I, which are made of spontaneous currents flowing on each bond of the RuO_4 plaquette inside the *ab*-plane of a RuO_6 octahedron (Supplementary Fig. 8b). We therefore focus on those loop currents of the type Θ_{II} which break inversion symmetry and are asymmetric because they consist of clockwise and

anticlockwise loop currents flowing only within selected areas of the RuO_4 plaquette, as illustrated in Supplementary Fig. 8c.

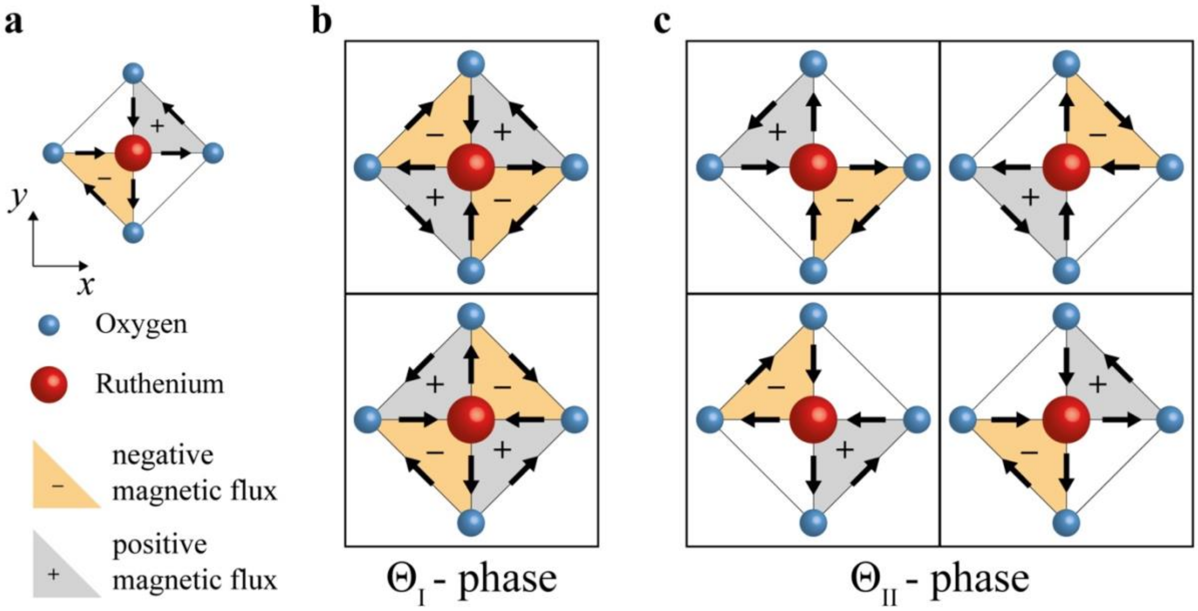

Supplementary Figure 8: Loop current phases. a, Illustration of the RuO_4 plaquette with example of orbital loop currents and magnetic fluxes generated. Loop currents flowing clockwise (anticlockwise) generate magnetic flux point inward (outward) the RuO_4 plane and they are labelled with an orange (grey) triangle with a '-' ('+') sign in the middle. **b, c,** Possible configuration of orbital loop current distributions with corresponding magnetic fluxes for a single RuO_4 plaquette of the type Θ_I (**b**) and of type Θ_{II} (**c**). Four RuO_4 plaquettes of either type Θ_I or of type Θ_{II} combine, along with a structural rotation, to give the total orbital current distribution for the SRO_{214} supercell as shown in Fig. 5c of the main text. Orbital loop current phases of type Θ_I are characterized by magnetic fluxes alternating in sign within the four triangles of the RuO_4 plaquette, and they break the C_4 transformation symmetry and time reversal symmetry, but they preserves inversion symmetry (e.g., the state is preserved after a rotation of $\pi/2$ about the centre followed by a reversal of the sign of the flux). These symmetry properties, however, make the type Θ_I phase not compatible with the inversion symmetry breaking expected on the SRO_{214} surface.

Given the reconstruction of the RuO_6 octahedra occurring in SRO_{214} near its surface, we restrict our analysis to those states combining, within a given SRO_{214} supercell, a loop current of the type Θ_{II} for each RuO_4 plaquette with the rotation of the same plaquette (see Fig. 5c of the main text). Within this configuration, we only consider states associated with staggered orbital fluxes. This is because states associated instead with a spontaneous flow of currents or with charge accumulation would not be physically compatible with the metallic state of SRO_{214}.

We adopt a microscopic description of the orbital loop current states having the features described above. Our microscopic model includes *d*-orbitals (t_{2g} orbitals) at Ru site and *p*-orbitals at planar O sites. We adopt a tight-binding description of the electronic configuration that includes Coulomb interactions both between the electron densities on Ru and O atoms and between electrons in the *p*-states on neighbouring O sites. The *d*-*p* and *p*-*p* Coulomb interactions are responsible for the electronic instabilities leading to the formation of the loop current phase. Our model includes the canonical $L \cdot s$ atomic spin-orbit coupling between the effective $L = 1$ angular

momentum representation for the t_{2g} sector of the d-orbitals and the s = 1/2 electron spin at the Ru site. The spin-orbit coupling interaction and its strength depend on the crystal field potential associated with the octahedral distortions (i.e., both flattening and rotations of the RuO_6 octahedra).

For the calculations, we adopt electronic parameters derived from ab-initio calculations (refs. [35,45] of the main text). The values that we use for the non-vanishing nearest neighbour hopping parameters t and the on-site orbital dependent energies ε are summarized in the Supplementary Table 1 below.

$t_{d_{xy}-p_y}$	$t_{d_{xz}-p_z}$	$t_{p_x-p_y}$	$t_{p_z-p_z}$	$\varepsilon_{d_{xy}}$	$(\varepsilon_{d_{xz}}, \varepsilon_{d_{yz}})$	ε_{p_x}	ε_{p_y}	ε_{p_z}
1.28 eV	1.28 eV	0.39 eV	0.15 eV	-2.34 eV	-2.36 eV	-4.62 eV	-4.52 eV	-4.51 eV

Supplementary Table 1: List of model parameters. Nearest neighbour hopping parameter amplitudes t for several pairs of orbitals and on-site orbital energies ε (orbitals are indicated as subindexes).

We write the model Hamiltonian in a compact form by introducing a basis vector for the unit cell that includes two inequivalent Ru atoms and four O atoms. Supplementary Fig. 9 shows the hybridization processes between p- and d- orbitals along with their signs defined by the orbitals' spatial symmetries. We identify two different possible electronic phases, here denoted as LC^+ and LC^- phase, which break time reversal symmetry due to the formation of orbital loop currents. The two phases differ in their orbital contributions, namely for the loop current distributions of the d_{xy} and (d_{xz}, d_{yz}) sectors, which are additive for the LC^+ state and cancelling for the LC^- state (see Fig. 5b, c of the main text).

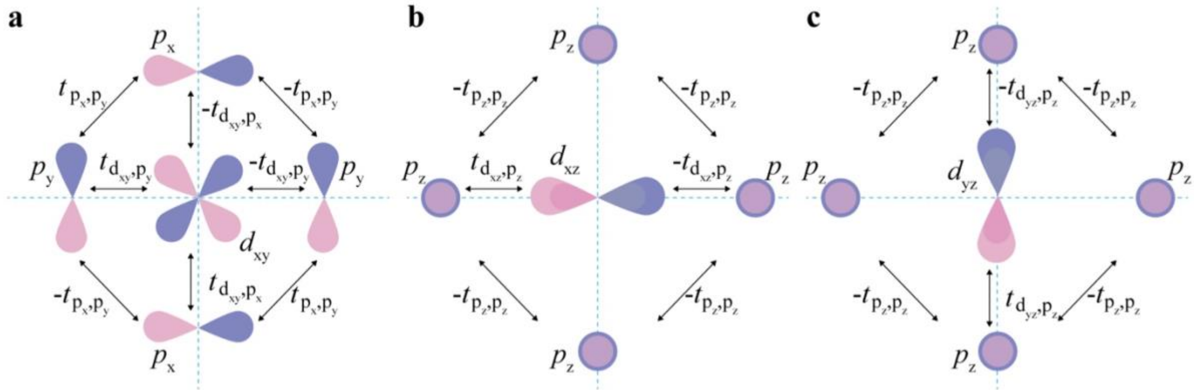

Supplementary Figure 9: Ru-O hybridization processes in SRO_{214}. a-c, Illustration of the Ru-O hybridization processes in SRO_{214} for the d_{xy} (**a**), d_{xz}(**b**) and d_{yz}(**c**) orbitals with corresponding hopping parameters t.

Determination of the ground state

In addition to investigating which is the most energetically favourable configuration between the LC^- and LC^+ state, we also study whether any loop current phases with non-zero magnetic fluxes have a lower free energy minimum compared to the conventional normal metal phase of SRO_{214} with zero magnetic fluxes.

To determine whether the LC$^-$ and LC$^+$ phase represents the most energetically favourable state (i.e., the ground state) of the SRO_{214} system, we decouple the inter-site Coulomb interaction in terms of the asymmetric bonding operator $\hat{\phi}_{\mathrm{lm}}^{\alpha\beta} = i\left(c_{\mathrm{l},\alpha}^{\dagger} c_{\mathrm{m},\beta} - c_{\mathrm{m},\beta}^{\dagger} c_{\mathrm{l},\alpha}\right)$ for the l-m bond between the atoms with positions identified by the coordinates a_{l} and a_{m} within the SRO_{214} unit cell. Here, $c_{l,\alpha}$ ($c_{\mathrm{l},\alpha}^{\dagger}$) are the annihilation (creation) operators for an electronic state with α orbital at the atomic site with coordinate a_{l}. The annihilation and creation operators $c_{\mathrm{m},\beta}$ and $c_{\mathrm{m},\beta}^{\dagger}$ are similarly defined for the electronic state associated with the β orbital at the atomic site with coordinate a_{m}.

The inter-site Coulomb interaction for a generic l-m bond (U_{lm}) fulfils the relation

$$U_{\mathrm{lm}} n_{\mathrm{l},\alpha} n_{\mathrm{m},\beta} = -\left(\frac{1}{2}\right) U_{\mathrm{lm}} \left(\hat{\phi}_{\mathrm{lm}}^{\alpha\beta}\right)^{\dagger} \hat{\phi}_{\mathrm{lm}}^{\alpha\beta} + \left(\frac{1}{2}\right) U_{\mathrm{lm}} \left(n_{\mathrm{l},\alpha} + n_{\mathrm{m},\beta}\right), \qquad \text{(Eq. S4)}$$

where $n_{\mathrm{l},\alpha} = c_{\mathrm{l},\alpha}^{\dagger} c_{\mathrm{l},\alpha}$ and $n_{\mathrm{m},\beta} = c_{\mathrm{m},\beta}^{\dagger} c_{\mathrm{m},\beta}$. By decoupling the interacting part of the quartic term, we introduce an order parameter ϕ associated with the expectation value of $\hat{\phi}_{\mathrm{lm}}^{\alpha\beta}$ and rewrite the interaction as

$$U_{\mathrm{lm}} n_{\mathrm{l},\alpha} n_{\mathrm{m},\beta} \cong$$
$$\cong -\left(\frac{1}{2}\right) U_{\mathrm{lm}} \left[\left(< \hat{\phi}_{\mathrm{lm}}^{\alpha\beta} > \left(\hat{\phi}_{\mathrm{lm}}^{\alpha\beta}\right)^{\dagger} + h.c \right) - \left| < \hat{\phi}_{\mathrm{lm}}^{\alpha\beta} > \right|^{2} \right] + \left(\frac{1}{2}\right) U_{\mathrm{lm}} \left(n_{\mathrm{l},\alpha} + n_{\mathrm{m},\beta}\right) \qquad \text{(Eq. S5)}$$

in which the term linear in density leads to a renormalization of the chemical potential. The average value $< \hat{\phi}_{\mathrm{lm}}^{\alpha\beta} >$ is evaluated by taking into account the contributions of all the Bloch states weighted by the corresponding Fermi distribution factor. In our analysis, to directly compare the free energies of the LC$^-$ and LC$^+$ phase we assume that $< \hat{\phi}_{\mathrm{lm}}^{\alpha\beta} > = \pm\phi$, where the positive or negative sign is taken according to the sense of circulation of the orbital loop currents shown in Fig. 5c of the main text.

We calculate the phase stability of the LC$^+$ and LC$^-$ configurations by determining the minimum of the free energy $E(\phi)$ with respect to the free energy of the normal state $E(0)$ as a function of the order parameter ϕ, which is associated with the amplitudes $< \hat{\phi}_{\mathrm{lm}}^{\alpha\beta} >$ within the SRO_{214} unit cell. The results of the free energy calculation in Fig. 5d of the main text show that the free energy of the LC$^+$ and LC$^-$ states $E(\phi)$ can be lower than $E(0)$ if U is sufficiently large. This result implies that, above a certain threshold of the inter-site Coulomb interaction U, the LC$^+$ and LC$^-$ phases indeed represent the ground state of the SRO_{214} system and they are therefore more energetically favoured compared to the normal state with no magnetic fluxes. Fig. 5d of the main text also shows

that the LC^- phase is more stable than the LC^+ phase, meaning the most stable orbital loop current phase consists of oppositely circulating currents that generate opposite magnetic fluxes in the *xy*- and *z*- orbital sectors. Iterative self-consistent calculations give variations of the amplitude of the loop current on each bond, which do not affect the quality of the final result, namely that the LC^- phase is the most energetically favoured.

Magnetic field generated by the orbital loop current phase

We calculate the magnetic field generated by the most stable orbital loop current phase LC^- and compare its magnitude with that measured experimentally by low-energy muons.

After determining $< \hat{\phi}_{\mathrm{lm}}^{\alpha\beta} >$, we compute the expectation value of the current operator on a given $l - m$ bond defined as $< \hat{J}_{\mathrm{lm}}^{\alpha\beta} > = \left(\frac{e}{\hbar}\right) f_{\mathrm{lm}}^{\alpha\beta} < \hat{\phi}_{\mathrm{lm}}^{\alpha\beta} >$, where $\hbar$ is the reduced Planck constant, e is the elementary charge, and $f_{\mathrm{lm}}^{\alpha\beta}$ is the effective energy associated with charge processes between α and β orbitals including both hopping and the symmetry breaking contributions arising from Coulomb interactions. We then determine the magnetic field generated by the average current $< \hat{J}_{\mathrm{lm}}^{\alpha\beta} >$ by using the Biot-Savart law, whilst taking into account the orbital dependent directions of the loop currents. For this calculation, we assume distances between Ru and O atoms of $d_{\mathrm{Ru-O}} \cong 1.9$ Å and $d_{\mathrm{O_x-O_y}} \cong 2.7$ Å (ref. [24] of the main text) and use the *d*-*p* and *p*-*p* hopping parameter amplitudes for the orbital dependent hybridization processes reported in Supplementary Table 1.

As indicated above, we restrict our analysis to the magnetic field generated by the orbital currents of the LC^- phase, since this is the ground state of the system. Consistently with the definition of the LC^- state, we determine the magnetic field assuming that the orbital currents in each Ru-O_x-O_y plaquette have opposite circulation directions. We note that, in this configuration, the loop currents flowing along the Ru-O_x and Ru-O_y bonds have a comparable effective kinetic energy $f_{\mathrm{lm}}^{\alpha\beta}$ and amplitude of $< \hat{\phi}_{\mathrm{lm}}^{\alpha\beta} >$ for the contributions associated with either the d_{xy} or the $(d_{\mathrm{xz}}, d_{\mathrm{yz}})$ orbitals, but opposite in sign so that the corresponding $< \hat{J}_{\mathrm{lm}}^{\alpha\beta} >$ terms tend to cancel out. The only orbital currents that do not cancel out are those flowing along the O_x-O_y bonds due to inequivalent hybridization processes for the *p*-orbitals. The resulting magnetic field generated by these unbalanced orbital currents is vanishingly small at the centre of a RuO_4 plaquette.

Since $< \hat{\phi}_{\mathrm{lm}}^{\alpha\beta} > \sim 10^{-1}$ for the minimum of the free energy of the LC^- state as the data in Fig. 5d of the main text show, and using $\mu_{\mathrm{m}} \cong 2 \cdot \mathrm{Gauss\ m\ Ampere}^{-1}$ for the SRO214 magnetic permeability μ_{m} (ref. [10] of the main text) and the $t_{\mathrm{p_x-p_y}}$ and $t_{\mathrm{p_z-p_z}}$ hopping parameter values

reported in Supplementary Table 1, we obtain an estimate of the magnetic field B generated by the orbital loop currents in the LC^- state within the range from 5 to 15 Gauss. The estimate of B is obtained under the assumption that the muons are implanted closer to an O atom (due to its higher electron affinity compared to Ru), which is at an average distance of ~ 2Å in SRO_{214} from the closest Ru (ref. [24] of the main text). The value we estimate for B is in agreement with the field strength determined from the experimental data in Fig. 4 of the main text of ~10 Gauss. We also note that the magnetic field generated by these loop currents can further decrease when approaching a critical value of U for which the net sum of the $< \hat{\phi}_{\mathrm{lm}}^{\alpha\beta} >$ contributions to the order parameter ϕ vanishes. The values of U for which ϕ vanish are calculated based on our model for both the LC^- and LC^+ states and shown in Supplementary Fig. 10.

The results in Supplementary Fig. 10 also indicate that, apart from a region very close to the critical U value, the order of magnitude of the induced magnetic field does not vary much as a function of U.

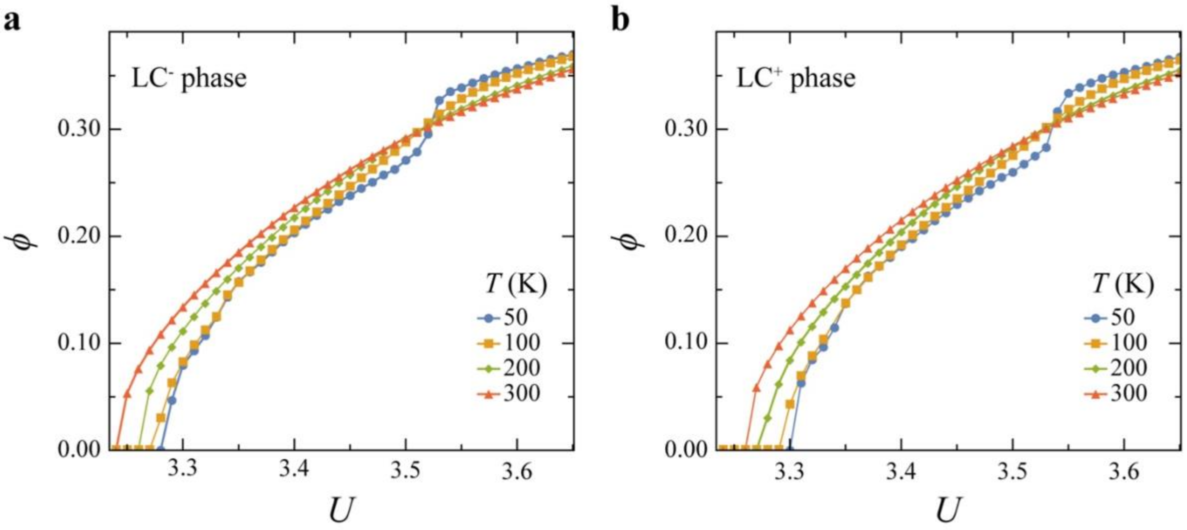

Supplementary Figure 10: Dependence of order parameter on the Coulomb repulsion. a, b, Evolution of the order parameter ϕ as a function of the Coulomb potential U for the LC^- (**a**) and LC^+ (**b**) states at different temperatures (indicated in the figures legends).
